# Supplementary material for: Fabrication of Carbon Nanomaterials Using Laser Scribing on Copper Nanoparticles-Embedded Polyacrylonitrile Films and Their Application in a Gas Sensor
Source: Polymers (Basel). 2021 Apr 28;13(9):1423. doi: 10.3390/polym13091423 (PMC8124524; doi:10.3390/polym13091423)
Supplement: Supplementary file 1 [file polymers-13-01423-s001.zip › polymers-1178794 - Supplementary Material-done.pdf]

polymers

MDPI

Supplementary Material

Fabrication of carbon nanomaterials using laser scribing on copper nanoparticles-embedded polyacrylonitrile films and their application in a gas sensor

Fabrication of Carbon Nanomaterials Using Laser Scribing on Copper Nanoparticles-Embedded Polyacrylonitrile Films and Their Application in a Gas Sensor

Yong-il Ko [1], Min-Jae Kim [1], Dong-Yun Lee [1], Jungtae Nam [1], A-Rang Jang [2,3,*], Jeong-O Lee [3] and Keun-Soo Kim [1,*]

[1] Department of Physics and Graphene Research Institute, Sejong University, Seoul 05006, Korea; armist130@gmail.com (Y.-i.K.); mjkimphys@gmail.com (M.J.K.); geovi012@gmail.com (D.Y.L.); goodnjt@kist.re.kr (J.N.)
[2] Department of Electrical Engineering, Semyung University, Jecheon 27136, Korea
[3] Advanced Materials Division, Korea Research Institute of Chemical Technology (KRICT), Gajeong-ro 141, Daejeon 34114, Korea; jolee@krict.re.kr
* Correspondence: arjang@semyung.ac.kr (A.-R.J.); kskim2676@sejong.ac.kr (K.S.K.); Tel.: +82-43-649-1303 (A.-R.J.); +82-2-3408-3316 (K.S.K.)

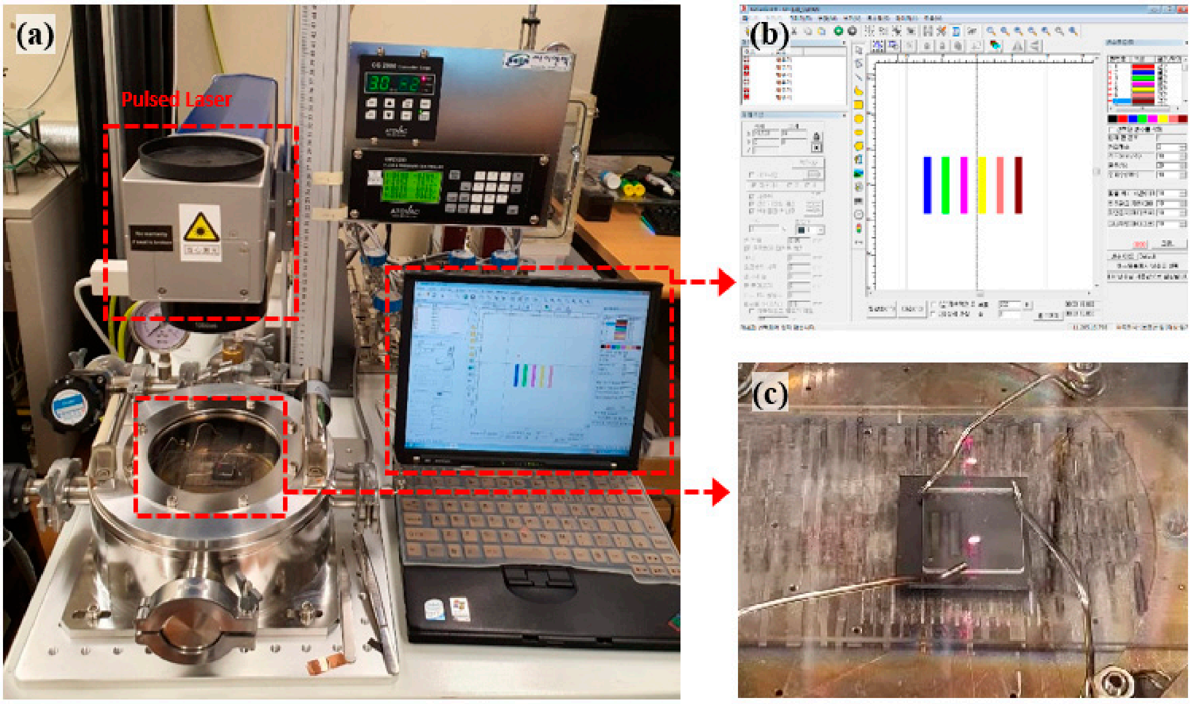

Figure S1. Photographs of (**a**) laser assisted CVD system, (**b**) operating software and (**c**) target sample in chamber (see the “Laser assisted CVD_chamber.mp4” video file).

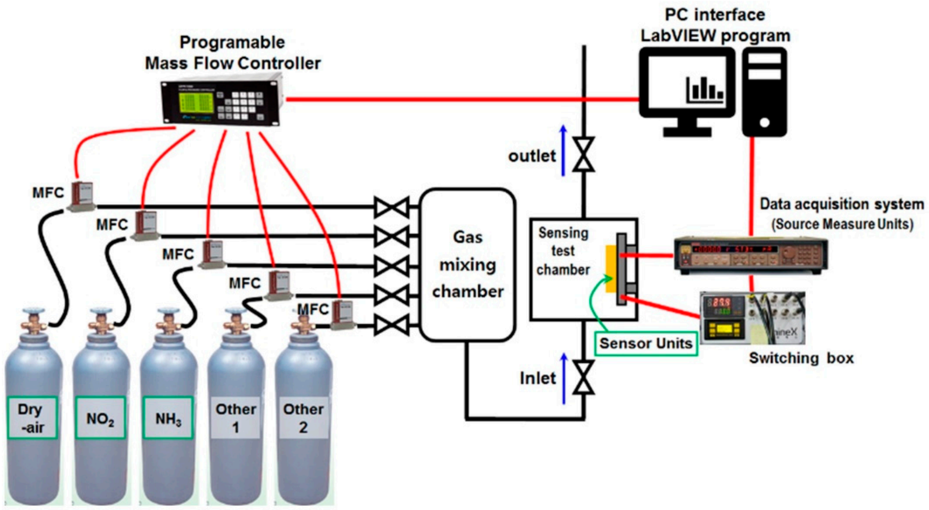

Figure S2. Schematic of the gas sensing system.

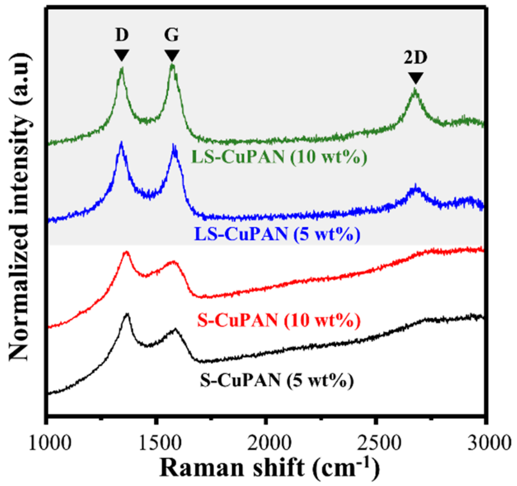

Figure S3. Raman spectra of S-CuPAN and LS-CuPAN with different weight ratios of Cu to PAN.

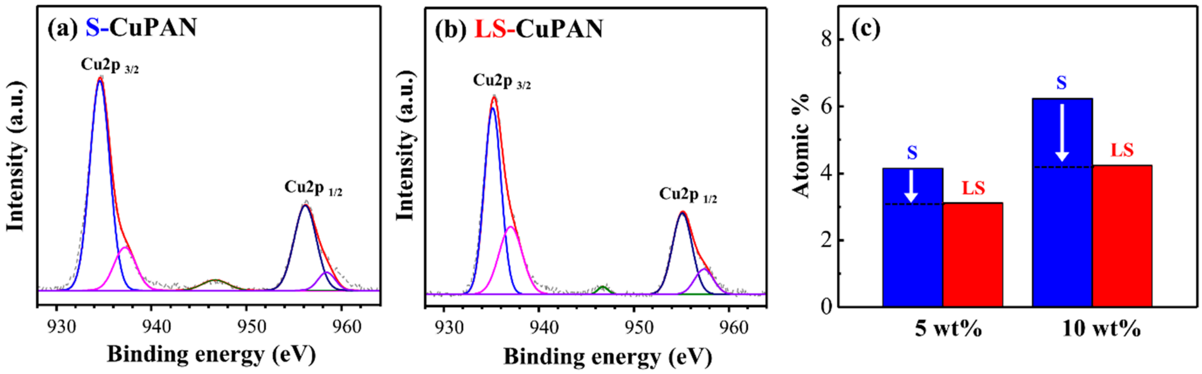

Figure S4. Cu2p spectra of (**a**) S-CuPAN (10 wt%), (**b**) LS-CuPAN (10 wt%), and (**c**) atomic ratio of Cu in XPS analysis.

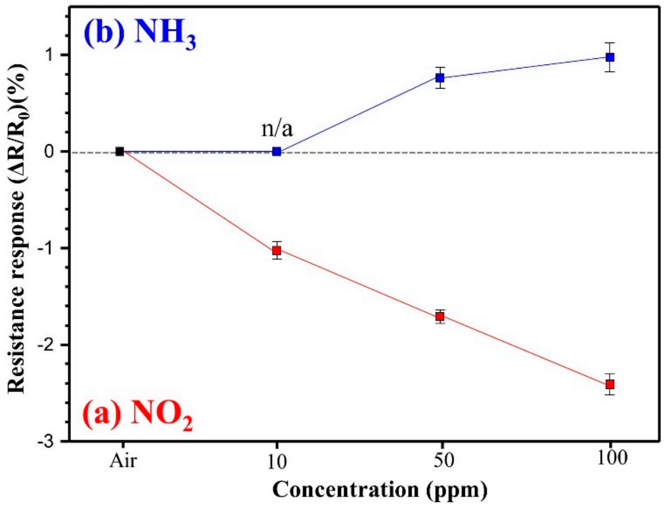

Figure S5. Resistance changing rates exposure to different gas concentrations, (**a**) under NO_2 10, 50 and 100 ppm, (**b**) under NH_3 10, 50 and 100 ppm.

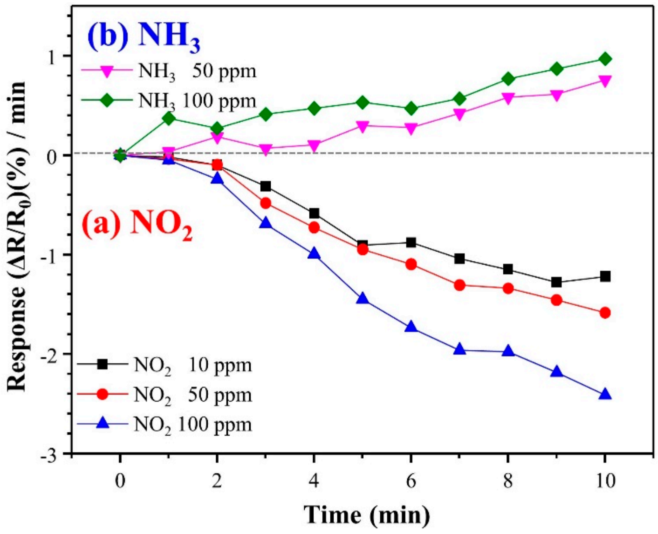

Figure S6. Resistance response versus time when (**a**) NO_2 and (**b**) NH_3 with different concentrations.
